# Characterizing Neurocognitive Impairment in Juvenile Fibromyalgia Syndrome: Subjective and Objective Measures of Dyscognition

**DOI:** 10.3389/fped.2022.848009

**Published:** 2022-02-24

**Authors:** Sabrina Gmuca, Maitry Sonagra, Rui Xiao, Elizabeth Mendoza, Kimberly S. Miller, Nina H. Thomas, Jami F. Young, Pamela F. Weiss, David D. Sherry, Jeffrey S. Gerber

**Affiliations:** ^1^Department of Pediatrics, Division of Rheumatology, Children's Hospital of Philadelphia, Philadelphia, PA, United States; ^2^Center for Pediatric Clinical Effectiveness, Children's Hospital of Philadelphia, Philadelphia, PA, United States; ^3^PolicyLab, Children's Hospital of Philadelphia, Philadelphia, PA, United States; ^4^Children's Hospital of Philadelphia, Perelman School of Medicine, University of Pennsylvania, Philadelphia, PA, United States; ^5^Seattle Children's Hospital, University of Washington, Seattle, WA, United States; ^6^Department of Biostatistics, Epidemiology and Informatics, Perelman School of Medicine at University of Pennsylvania, Philadelphia, PA, United States; ^7^Milken Institute School of Public Health, The George Washington University, Washington, DC, United States; ^8^Center for Human Phenomic Science Behavioral Neuroscience Core, Children's Hospital of Philadelphia, Philadelphia, PA, United States; ^9^Department of Child and Adolescent Psychiatry and Behavioral Services, Children's Hospital of Philadelphia, Philadelphia, PA, United States; ^10^Department of Pediatrics, Division of Infectious Diseases, Children's Hospital of Philadelphia, Philadelphia, PA, United States

**Keywords:** dyscognition, juvenile fibromyalgia syndrome, pediatrics, pediatric chronic pain, musculoskeletal pain and adolescents

## Abstract

**Objectives:**

Our understanding of brain fog, or dyscognition, among youth with juvenile fibromyalgia syndrome is limited. We aimed to determine the prevalence of subjective (self-reported) and objective dyscognition, as well as factors associated with subjective dyscognition in juvenile fibromyalgia syndrome.

**Methods:**

A cross-sectional cohort study of patients (*n* = 31) 12-17 years old diagnosed with primary juvenile fibromyalgia syndrome and one of their parents from 2017 to 2019. Subjects completed a series of survey measures and patients completed a brief neurocognitive battery. Subjective dyscognition was determined based on scores on the Pediatric Quality of Life Inventory (PedsQL) Cognitive Functioning Scale and Behavior Rating Inventory of Executive Function (BRIEF-2) global executive composite (GEC). Objective dyscognition was defined as impairment of more than two standard deviations in any of the neurocognitive domains. We used Fisher's exact test or Wilcoxon rank-sum test, as appropriate, to compare clinical patients based on the presence of dyscognition. Multivariable logistic regression modeling was performed to determine factors associated with subjective dyscognition.

**Results:**

Of the 31 subjects, 65% reported subjective dyscognition and 39% had objective dyscognition, primarily in the domains of psychomotor speed (23%), executive function (23%), and attention (3%). Subjective dyscognition was not indicative of objective dyscognition. Subjective dyscognition was independently associated with functional disability (OR: 1.19 [95% CI: 1.02-1.40]) and anxiety (OR: 1.12 [95% CI: 1.02-1.24]).

**Discussion:**

Adolescents with fibromyalgia predominantly experience subjective dyscognition but more than 1/3 also experience objective dyscognition. Future research should explore the impact of interdisciplinary rehabilitation programs on the treatment of dyscognition in youth with JFMS.

## Introduction

Approximately 50% of adults with fibromyalgia report cognitive impairment (referred to as brain fog or “fibro fog”) including difficulty in ability to attend, concentrate, remember, use language, multitask, and organize information (1, 2). This co-morbid neuropsychological symptom pattern in adults with fibromyalgia has been termed cognitive dysfunction or dyscognition, which includes loss of mental clarity and problems with attention and memory (3–6). Dyscognition in fibromyalgia encompasses objective cognitive difficulties on neuropsychological tests and also self-reported cognitive complaints (7, 8). From previous studies in adults with fibromyalgia syndrome, however, it is unclear whether patients' self-reported dyscognition is subjective or if they, in fact, experience objective dyscognition as measured by neurocognitive testing (3–5, 9, 10).

Dyscognition in adults with fibromyalgia has been identified in five cognitive domains: executive function, working memory, semantic memory, episodic memory, and attention (8, 11). Executive function is a broad term referring to functions mediated by the frontal lobe (3). Working memory is involved in storing and processing information and the ability to multitask. Attention allows the brain to select the relevant inputs for storage and processing into working memory. Semantic memory is the knowledge of words or facts and episodic memory is the ability to recall and mentally re-experience specific episodes from one's personal past. Many factors may contribute to a patient's neurocognitive function including resilience, depression, anxiety, sleep, fatigue, and pain. Therefore, these factors should be formally assessed when evaluating an individual's cognitive functioning.

Dyscognition has numerous socioeconomic and psychosocial sequelae in adults with fibromyalgia including functional disability, unemployment, and increased healthcare utilization (12, 13). These consequences are of even greater importance in children because dyscognition can contribute to school absenteeism, academic problems, and over-medicalization (13–15). Juvenile fibromyalgia syndrome (JFMS) is a pediatric non-inflammatory musculoskeletal pain syndrome characterized by widespread pain for ≥3 months accompanied by a host of somatic complaints (4, 16, 17). There is, however, a paucity of research systematically examining dyscognition in patients with juvenile fibromyalgia syndrome (18).

We aimed to determine the prevalence of subjective and objective dyscognition, and association between subjective and objective dyscognition in patients with primary JFMS. We hypothesized that similar to adults with fibromyalgia syndrome, youth with fibromyalgia would have a high prevalence of subject dyscognition ranging from 50 to 75% and that rates of objective dyscognition would be lower but substantial (>25%). We also hypothesized that subjective dyscognition (measured *via* self-report questionnaires) would not be indicative of objective dyscognition (measured *via* a battery of neurocognitive testing). A secondary objective of this study was to explore factors associated with subjective dyscognition in JFMS. We hypothesized that patients with greater co-morbid anxiety and depression would be more likely to endorse subjective dyscognition.

## Materials and Methods

### Study Population

This was a cross-sectional cohort study conducted at a tertiary care pediatric hospital from July 1, 2017 until October 1, 2019. The study population consisted of adolescents who were 12-17 years old and diagnosed with JFMS according to the 2010 American College of Rheumatology criteria for fibromyalgia syndrome (19) and one of their parents/legal guardians. Patients were evaluated in either the general pediatric rheumatology clinic or pediatric rheumatology pain clinic (goal evaluable *n* = 30; see power and sample size calculations below). The study received approval from our respective institutional review board. Enrolled patients and parents received compensation for their time and effort.

#### Inclusion Criteria

Included patients received a primary or provisional diagnosis of JFMS [in accordance with the 2010 American College of Rheumatology Fibromyalgia Syndrome criteria for adults (19)] by a physician at the time of their (1) initial consultation in either the general pediatric rheumatology clinic or the subspecialty pediatric rheumatology pain clinic, or (2) follow-up visit in the pediatric rheumatology pain clinic. Patients and their proxies were required to read or understand English well enough to complete study assessments, proxies had to provide permission (informed consent) for both self and child, and children needed to assent or consent to participation.

#### Exclusion Criteria

We excluded patients and/or proxies whose current medical status or cognitive functioning precluded completion of the assessment instruments as determined by the principal investigator (PI). Specific examples included (but were not limited to) traumatic brain injury, concussion or other head trauma, cerebral palsy or other chronic neurologic deficit, legally blind, hearing impairment or loss, epilepsy or other seizure disorder, systemic lupus erythematosus, auto-inflammatory brain disease (e.g., central nervous system vasculitis), intellectual disability (e.g., Down's syndrome, developmental delay) and/or autism spectrum disorder. We also excluded subjects who had one or more active prescriptions for a stimulant medication or did not have a legal guardian available to provide consent and/or complete study measures. These exclusion criteria were applied to minimize any confounding medical conditions that might affect cognitive functioning while also not being too restrictive. Study staff members were informed and encouraged to discuss with the PI any potential subjects to confirm the subjects met the eligibility criteria.

#### Ethics Statement

This study involving human participants was reviewed and approved by the Children's Hospital of Philadelphia Institutional Review Board. The patients/participants provided their written informed consent to participate in this study.

### Study Procedures

#### Data Collected as Part of Routine Clinical Care

Demographics and clinical data collected as part of routine clinical care were abstracted from the electronic medical record for all patients. This included the following: physical exam, vital signs, previous laboratory or imaging results and documentation of current and past medications as well as the following patient-reported outcome measures:

*The Functional Disability Inventory (FDI)* (20–22) is a 15-item measure assessing the degree to which pain interferes with children's physical and psychosocial functioning over a 2-week period by both child- and parent-report. Higher scores (0-60) indicate greater functional disability. The FDI has demonstrated adequate psychometric properties including reliability and validity with youth ages 8-18 years with chronic musculoskeletal pain. Minimal clinically important difference (MCID) for this measure is a 7.8 point reduction (23) and the score can be categorized into 4 levels of disability: no/minimal (0-12); mild (13–20); moderate (21–29); and severe (≥30) (21, 24).

*The Visual Analog Pain Scale (VAS)* (25) is a child-report measure assessing pain intensity. Children are given a 100 mm visual analog scale and asked to indicate their pain intensity, with 0 = “no pain” and 100 = “worst pain imaginable” by drawing a mark along the scale. VAS pain intensity ratings have established reliability and validity across many pediatric populations. Minimal clinically important difference (MCID) is a 1-point reduction (26).

*The Widespread Pain Index (WPI)* (19, 27, 28) is a component of the 2010 American College of Rheumatology Criteria for Fibromyalgia Syndrome. Patients report each of the pre-designated areas of the body where they have experienced pain over the past 7 days, taking into account their current therapies and treatments, and excluding pain or symptoms from other known illnesses. The WPI score ranges from 0 to 19 (with each body region affected receiving one point) with higher values indicating greater involvement of different anatomical regions.

*The Symptom Severity Scale (SSS)* (19, 27, 28) is a questionnaire that assesses subjects' level of symptom severity across three domains (fatigue; waking unrefreshed; cognitive symptoms) and the extent (severity) of somatic symptoms in general. Scores total between 0 and 12 with higher scores indicating greater severity. In order to meet criteria for fibromyalgia syndrome, a patient must have a WPI ≥ 7 and SSS ≥ 5 or WPI 3-6 and SSS ≥ 9 (in addition to having symptoms for at least 3 months and the lack of a disorder that would otherwise explain the pain).

While designed for an adult population, the 2010 ACR criteria for fibromyalgia syndrome [including both the widespread Pain Index (WPI) and symptom severity scale (SSS)] have demonstrated validity in teenage females who comprise most of the youth with JFMS with very good sensitivity (89%) and specificity (88%). Therefore, while imperfect, they are the only criteria for fibromyalgia that have been analyzed and validated for a pediatric population (29, 30). There are no established data on reliability or the MCID for the WPI or SSS.

### Study Measures

#### Questionnaires

At the time of the study visit both patients and their consented parents completed a series of questionnaires. All the following measures were patient self-report and parent-proxy child report, except for the Resilience Scale 14-item (RS-14), which was completed as a self-report for both patients and parents. Among the following questionnaires, PedsQL multidimensional fatigue scale, PROMIS pediatric global health (PGH-7) measure, and RS-14 scale were completed by the patient-proxy pair on a smart tablet upon arrival in one of the family conference rooms. The remaining measures were administered by an experienced psychometrist under the supervision of a neuropsychologist during the same visit, but in a different location/room. We describe reliability, validity and MCID when appropriate for each of the measures. For more detailed information please see the cited references.

*The Patient-Reported Outcomes Measurement Information System (PROMIS) Pediatric Global Health (PGH-7) Measure* (31, 32) is a summary assessment of a child's health representing an individual's overall assessment of their health, focusing on physical, mental and social health components (33). Measure reliability is excellent. It was assessed using internal consistency alpha and intraclass correlation coefficients from a test-retest sample. The internal consistency alpha for the child sample was 0.88 and 0.84 for the parent sample. The test-retest reliability coefficients were 0.73 for child report and 0.74 for parent- proxy report. The measure has structural and content validity has demonstrated excellent convergent and discriminant validity with other PROMIS pediatric measures. Raw scores are converted to T-score values with a mean score of 50 (standard deviation of 10). MCID for this measure is 3.0 (33).

*The Children's Depression Inventory, 2nd Edition (CDI-2)* (34) is a standardized, 28-item assessment of depressive symptoms in children ages 7-17 years. T-scores ≥ 65 identify potentially clinically depressed individuals. The CDI-2 has demonstrated adequate to excellent psychometric properties and is sensitive to changes in depressive symptoms over time. Internal consistency for the CDI-2 self-report is supported by alpha coefficients ranging from 0.67 to 0.91 and good test-retest reliability. Construct validity and discriminant validity have also been established.

*The Multidimensional Anxiety Scale for Children, 2nd Edition (MASC-2)* (35–37) is a standardized, 50-item questionnaire assessing anxiety symptoms in youth aged 8-19 years. Good internal consistency, discriminant validity, and test-retest reliability have been established. T-scores ≥ 60 indicate increased likelihood of at least one anxiety disorder in the subject.

*Behavior Rating Inventory of Executive Function, 2nd Edition (BRIEF-2)* *(**38**)* is a standardized rating scale used to assess children's executive functions in home and school environments. The BRIEF-2 Self-Report Form is a 55-item standardized self-report measure developed to capture older children's and adolescents' (aged 11-18 years with a fifth-grade or better reading level) views of their own executive functions, or self-regulation, in their everyday environment. The BRIEF-2 Self-Report Form was intended to complement the 63-item BRIEF-2 Parent form in order to meet the need for capturing adolescent's views of their self-regulatory strengths and weaknesses. For all BRIEF-2 clinical scales and indexes, T scores from 60 to 64 are considered mildly elevated, and T scores from 65 to 69 are considered potentially clinically elevated.

*The (PedsQL™, Copyright* ©*1998 JW Varni, Ph.D. All rights reserved) Multidimensional Fatigue Scale (MFS)* (39–43) evaluates fatigue in children and adolescents through self- and proxy-reports. The scale assesses multidimensional fatigue through three dimensions/subscales: general (*The PedsQL General Fatigue scale*), sleep/rest (*The PedsQL Sleep/Rest Fatigue scale*), and cognitive fatigue (*The PedsQL Cognitive Functioning scale*) (39, 44, 45). Each subscale consists of a 6-item questionnaire that have been utilized across numerous pediatric chronic conditions, demonstrating significant correlations with the BRIEF-2 in a pediatric head trauma population (39, 44, 45). The questions are answered on a five-point Likert scale (transformed from 0 to 100), with higher scores indicating better health related quality of life (or indicating fewer problems). The PedsQL Multidimensional Fatigue Scale evidenced excellent feasibility, excellent reliability for the Total Scale Scores (patient self-report α = 0.90; parent proxy-report α = 0.95), and acceptable reliability for the three individual scales (patient self-report α = 0.77–0.84; parent proxy-report α = 0.90–0.97) in a population of patients with another pain condition, sickle cell disease (40).

*The 14-Item Resilience Scale* (46–49) (©2009 Gail M. Wagnild and Heather M. Young. Used by permission. All rights reserved. “The Resilience Scale” is an international trademark of Gail M. Wagnild and Heather M. Young, 1993) is a well-validated questionnaire consisting of 14 items to assess resilience. Scores on this scale range from 14 to 98 with a higher score indicating greater resilience and it has been used for over 25 years in variety of individuals of different ages (including adolescents), socioeconomic, and educational backgrounds although data supporting its use for the younger pediatric population are limited (48, 50, 51). The scale focuses on domains of resilience including purpose, perseverance, self-reliance, equanimity and authenticity. A higher score indicates greater resilience. Specifically, scores below 65 indicate low resilience, between 65 and 81 show moderate resilience, and above 81 include high resilience. The RS-14 has demonstrated internal consistency reliability with Cronbach's alpha coefficients ranging from 0.84 to 0.94.

### Neurocognitive Testing

Patients completed an abbreviated neurocognitive battery assessing the domains outlined in Table 1. The battery of neurocognitive tests was conducted on the single study visit date, totaled ~2-3 h, and was administered by an experienced psychometrist under the supervision of a neuropsychologist, who collected the data following a standardized protocol. For each of the listed measures, we describe reliability, validity and MCID when appropriate. For more detailed information please see the cited manuals.

**Table 1:** Battery of testing to assess neurocognitive function.

**Cognitive domain**	**Test**
General intellect	Wechsler Abbreviated Scale of Intelligence Second Edition (WASI-II)
Memory	• Children and Adolescent Memory Profile (ChAMP) • Digit Span subtest from either the Wechsler Intelligence Scale for Children—Fifth Edition (WISC-V) or Wechsler Adult Intelligence Scale—Fourth Edition (WAIS-IV), depending on subject's age
Attention	Conners Continuous Performance Test 3rd Edition (CPT-3)
Executive function	Delis-Kaplan Executive Function System (D-KEFS): Verbal Fluency Test, Trail Making Test, and Color-Word Interference Test
Complex psychomotor speed	Grooved Pegboard Test

*The Wechsler Abbreviated Scale of Intelligence (WASI-II)* (52, 53) is an abbreviated measure of cognitive intelligence (ages 6-89 years). It is an instrument that measures an individual's verbal, non-verbal and general cognitive functioning and has excellent internal consistency and test-retest reliability.

*The Children and Adolescent Memory Profile (ChAMP)* (54) is a comprehensive norm-referenced memory test, assessing both visual and verbal memory, for use with individuals ages 5 through 21 years. Two verbal (i.e., Lists and Instructions) and two visual (i.e., Objects and Places) subtests contribute to six indices: Total Memory, Verbal Memory, Visual Memory, Immediate Memory, Delayed Memory, and Screening.

*The Digit Span subtest* (55–57) was performed from either the Wechsler Intelligence Scale for Children—Fifth Edition (WISC-V) or Wechsler Adult Intelligence Scale—Fourth Edition (WAIS-IV), depending on the subject's age, to test working memory ability. Children > 16 years of age were administered the Digit Span from the WAIS-IV and ≤16 years old received the version from the WISC-V. In the Digit Span subtest children are verbally given sequences of numbers and asked to repeat them, either as heard, in reverse order, or sequenced from smallest to largest. The subjects' span is the longest number of sequential digits that can accurately be remembered in each condition.

*The Conners Continuous Performance Test Third Edition (CPT-3)* (58) measures attention-related problems in individuals aged ≥ 8 years. By indexing the respondent's performance in areas of inattentiveness, impulsivity, sustained attention, and vigilance, the CPT-3 can aid in the assessment of Attention-Deficit/Hyperactive Disorder (ADHD) and other neurological conditions related to attention.

*Delis-Kaplan Executive Function System (D-KEFS)* (59–61) is a neuropsychological test used to measure a variety of verbal and non-verbal executive functions for both children and adults (aged 8-89 years). It comprises nine tests that were designed to stand alone (there are no aggregate measures or composite scores for an examinee's performance). The subtests employed in this study included the Verbal Fluency Test, Trail Making Test, and Color-Word Interference Test. The Verbal Fluency Test measures letter fluency, category fluency, and category switching. The Trail Making Test measures flexibility of thinking on a visual-motor sequencing test. The Color-Word Interference Test measures ability to inhibit a dominant and automatic verbal response. The D-KEFS helps determine how deficits in higher order thinking may impact an individual's functioning.

*The Grooved Pegboard Test* (62–64) assesses visual-motor coordination, motor speed and fine motor control. This manipulative dexterity test contains 25 holes with randomly positioned slots and pegs which have a key along one side. Pegs must be rotated to match the hole before they can be inserted. This procedure measures performance speed in a fine motor task by examining both sides of the body, inferences may be drawn regarding possible lateral brain damage.

### Other Study Measures

The *Memory Validity Profile (MVP)* (co-normed with ChAMP) was used along with the *Medical Symptom Validity Test (MVST)* (65, 66) to ensure subjects were not performing poorly due to poor effort. The MVP is a performance validity test specifically designed for, nationally standardized on, and validated for use with children, adolescents and young adults 5-21 years of age. In a study conducted during development, MVP cutoff scores had 100% sensitivity and 100% specificity in detecting feigned memory impairment. The MSVT is a short-computerized verbal memory screening test with multiple subtests measuring memory and response consistency. Performance at or below a score of 85 indicates a failure of the symptom validity subtests.

### Statistical Analysis

The outcome of subjective dyscognition (binary) was defined as a Pediatric Quality of Life Inventory (PedsQL) Cognitive Functioning Scale score ≤ 50 or self-reported Behavior Rating Inventory of Executive Function (BRIEF-2) global executive composite (GEC) score ≥ 70. Objective dyscognition (binary) was defined as impairment of more than two standard deviations (SD) in any of the domains in the neurocognitive battery. We assessed the association of clinical signs and symptoms with the presence of subjective and objective dyscognition using Fisher's exact test or Wilcoxon rank-sum test, as appropriate. The association between subjective and objective dyscognition was analyzed via Fisher's exact test. We assessed patient-proxy agreement using intra-class correlation coefficients (ICCs) and mean differences between patient and proxy scores on patient-reported outcome measures using Wilcoxon signed-rank test. Bivariate logistic regression or exact logistic regression was used to evaluate the marginal association of each factor with subjective dyscognition. Factors with *p*-value < 0.20 were considered in subsequent multivariable logistic regression. Stepwise selection algorithm was used to determine the final multivariable model. For all analyses regarding objective dyscognition, subjects with poor effort based on the Memory Validity Profile (MVP) or Medical Symptoms Validity Test (MSVT) were excluded. The data analyses for this study were performed using SAS 9.4 (Copyright 2002-2012 by SAS Institute Inc., Cary, NC, USA).

#### Sample Size and Power Calculations

Since the primary objective of this study was descriptive, we calculated sample size and power based on the secondary objective. Assuming the prevalence of subjective dyscognition in subjects with JFMS is 0.70, when the predictor is equal to its mean value, with *N* = 30 and 80% power at a significance level of 0.05 we determined we would be able to detect a change of this prevalence from 0.70 to 0.88 when the predictor increases by one SD from the mean. This change corresponds to an odds ratio (OR) of 3.1. We therefore aimed to recruit a total of at least 30 evaluable subjects for this study to be adequately powered for the primary study objective.

## Results

**Figure 1:**
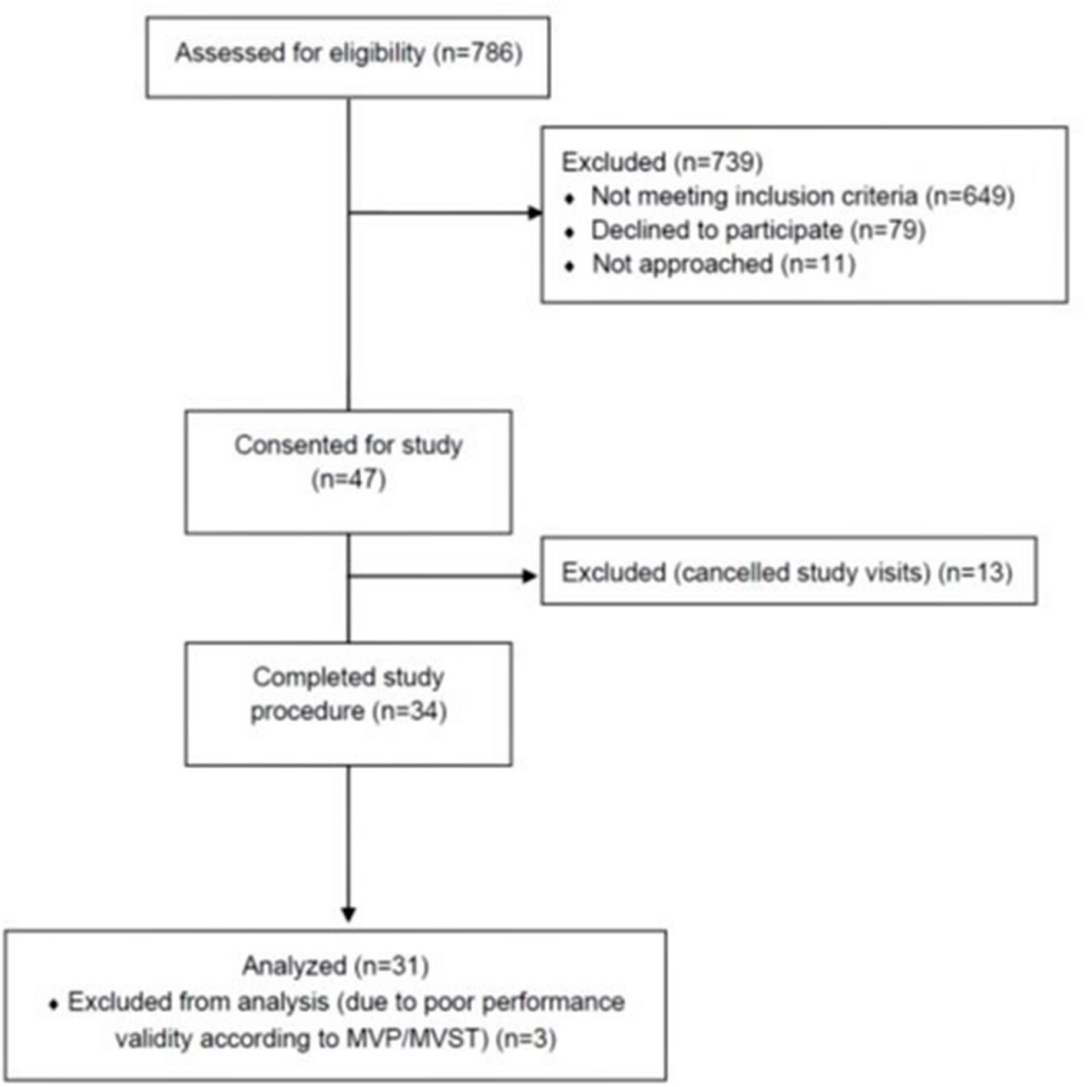
Flow chart of participant recruitment. MVP, memory validity profile; MVST, medical symptom validity test.

A total of 34 subjects met inclusion criteria and completed the study (Figure 1). Three subjects were excluded from analyses due to poor performance validity on the MVP/MVST (one of whom also deviated from the study protocol), resulting in 31 evaluable subjects. Table 2 shows patient demographics, clinical characteristics, and results from patient-reported outcome measures, stratified by the presence or absence of subjective dyscognition. Overall, most subjects were female (87%), non-hispanic (90%) and Caucasian (81%). Median age at enrollment was 15 years (IQR: 14-16). Fifteen patients received care in the pediatric rheumatology pain clinic, whereas 16 patients received care in the general pediatric rheumatology clinic. Patients reported prolonged pain, with a median pain duration of 12 months [IQR: 6-36]. Their pain tended to be widespread (median WPI = 11 [IQR: 9-13]) and accompanied by somatic complaints (median SSS=8 [IQR: 7-9]). Pain intensity was moderate with a median VAS score of 59 [IQR: 32-68]). Depression and anxiety were common with 45% of patients having clinical depression and 65% experiencing clinical anxiety according to their CDI-2 and MASC-2 scores, respectively.

**Table 2:** Demographics, clinical characteristics and patient-reported outcome measures (PROs) among adolescents with juvenile fibromyalgia syndrome.

	**All subjects** **(*n* = 31)**	**(+) Subjective** **dyscognition** **(*n* = 20)**	**(-) Subjective** **dyscognition** **(*n* = 11)**	***P*-value**
**Demographics**				
Age (median, IQR)	15 (14-16)	15 (14-16)	15 (13-16)	0.90
Female, *n* (%)	27 (87%)	17 (63%)	10 (37%)	0.40
White, *n* (%)	25 (81%)	16 (64%)	9 (36%)	0.31
Non-hispanic, *n* (%)	28 (90%)	18 (64%)	10 (36%)	0.46
**Clinical characteristics, median, IQR**				
Pain duration (months)	12 (6-36)	21 (12-36)	10 (5-60)	0.44
Pain visual analog scale (VAS) (0-100)	59 (32-68)	60 (52.5-67.5)	52 (16-68)	0.23
Widespread pain index (WPI) (0-19)	11 (9-13)	11 (8.5-13)	12 (9-14)	0.54
Symptom severity score (SSS) (0-12)	8 (7-9)	9 (7-9.5)	7 (5-9)	0.05
**Patient-reported outcome measures (PROs), median (IQR)**				
Functional disability inventory (FDI) (0-60)	23 (15-31)	25.5 (17-33)	16 (10-26)	0.02^I^
HRQOL [PROMIS global health 7 (PGH-7)]	38.8 (30.8-40.4)	36.4 (30-38.8)	40.4 (38.8-45.7)	0.01^I^
14-item resilience scale (14-98)	72 (59-81)	66 (53-75)	76 (72-86)	0.02^I^
CDI-2 (depression)	64 (51-72)	67 (55-76)	55 (47-59)	0.02^I^
MASC-2 (Anxiety)	65 (52-80)	70 (63.5-82)	52 (49-62)	0.01^I^
PedsQL general fatigue	33 (25-50)	25 (19-37.5)	50 (42-63)	<0.01^I^
PedsQL sleep/rest fatigue	42 (25-54)	40 (25-46)	54 (29-63)	0.06

I *Significant p-values suggesting statistical significance*.

*For subjective dyscognition, 20 subjects were positive on the PedsQL (Pediatric Quality of Life Inventory) Cognitive Functioning Scale only and eight subjects were positive on the BRIEF-2 GEC and PedsQL Cognitive Functioning Scale. IQR, interquartile range; Pain VAS, Pain visual analog scale where higher scores indicate more pain; SSS, Symptom Severity Scale, higher scores indicating greater symptom severity; WPI, widespread pain index, higher values indicating greater involvement of different anatomical regions where the child has experienced pain over the past 7 days; FDI, Functional disability inventory, greater scores indicating more functional disability; HRQoL, Health Related Quality of Life assessed using the PROMIS Pediatric Global Health 7 (PROMIS PGH-7). 14-item Resilience Scale ranges from 14 to 98, with greater scores indicating greater resilience. CDI-2, The Children's Depression Inventory, 2nd Edition where higher score (T-score ≥ 65) indicative of clinical depression. MASC-2, Multidimensional Anxiety Scale for Children, 2nd Edition, higher score (T-scores ≥ 60) indicate increased likelihood of at least one anxiety disorder in the subject. PedsQL (The Pediatric Quality of Life Inventory) General Fatigue and Sleep/Rest Fatigue scales are scored 0-100, where higher scores indicate less symptoms/problems in a dimension*.

Sixty-five percent (*n* = 20) of subjects reported subjective dyscognition. The median score on the BRIEF-2 Global Executive Composite (GEC) score was 58 (IQR: 50-71) and the median score on the PedsQL Cognitive Functioning Scale was 42 (IQR: 25-67). Of those who had subjective dyscognition (*n* = 20), 40% had abnormal scores on the BRIEF-GEC and 100% had abnormal scores on the PedsQL Cognitive Functioning Scale. Table 3 shows patient-proxy agreement on measures of symptom severity in the cohort. Patient-proxy agreement on mental health and neuropsychological symptoms was good to excellent (ICC ranging from 0.60 [95% CI: 0.19-0.80] to 0.86 [95% CI: 0.71-0.93]) and therefore we focused on the patient self-report for the remaining analyses.

**Table 3:** Patient-proxy agreement on measures of symptom severity in JFMS (*n* = 31).

**Variables (mean, SD)**	**Patient** **(*N* = 31)**	**Proxy** **(*N* = 31)**	**Patient-proxy differences**	**ICC (95% CI)**
Functional disability (FDI)	22.45 (9.95)	22.10 (9.42)	0.35 (6.83)	0.86 (0.71-0.93)
Multidimensional fatigue (PedsQL total MFS)	41.03 (17.23)	38.55 (18.35)	2.48 (13.32)	0.84 (0.66-0.92)
General fatigue (PedsQL General fatigue)	37.38 (20.50)	36.58 (18.07)	0.81 (15.80)	0.80 (0.59-0.91)
Sleep (PedsQL sleep)	41.19 (19.19)	35.94 (19.99)	5.26 (14.88)	0.82 (0.62-0.91)
Cognitive fatigue (PedsQL cognitive functioning)	44.45 (23.85)	43.32 (28.95)	1.13 (20.52)	0.83 (0.64-0.92)
Executive functioning (BRIEF-2 GEC T score)	59.13 (11.58)	59.13 (10.89)	0 (11.13)	0.68 (0.33-0.85)
Depression (CDI-2)	62.32 (12.52)	64.42 (13.63)	−2.10 (9.66)	0.84 (0.67-0.92)
Anxiety (MASC-2)	64.90 (14.70)	68.45 (15.52)	−3.55 (16.04)	0.60 (0.19-0.80)

*Functional disability inventory (FDI) scores range from 0 to 60 with greater scores indicating more functional disability. Pediatric Quality of Life Inventory (PedsQL) Multidimensional Fatigue Scale (MFS) and the subscales range from 0 to 100 where higher scores indicate less symptoms/problems. Behavior Rating Inventory of Executive Function-2 (BRIEF-2) is a standardized rating scale used to assess children's executive functioning where T scores from 60 to 64 are considered mildly elevated, and T scores from 65 to 69 are considered potentially clinically elevated. The Children's Depression Inventory, 2nd Edition (CDI-2) is an assessment of depressive symptoms, where T-scores ≥ 65 identify potentially clinically depressed individuals. The Multidimensional Anxiety Scale for Children, 2nd Edition (MASC-2) assesses anxiety symptoms in youth where T-scores ≥ 60 indicate increased likelihood of at least one anxiety disorder in the subject. Difference between patient and proxy mean scores were assessed with the Wilcoxon signed-rank test (two tailed). Intra-class correlation coefficients (ICCs) were rated as follows: poor agreement (≤0.40), fair agreement (0.41-0.59), good agreement (0.60-0.74), and excellent agreement (≥0.75)*.

**Figure 2:**
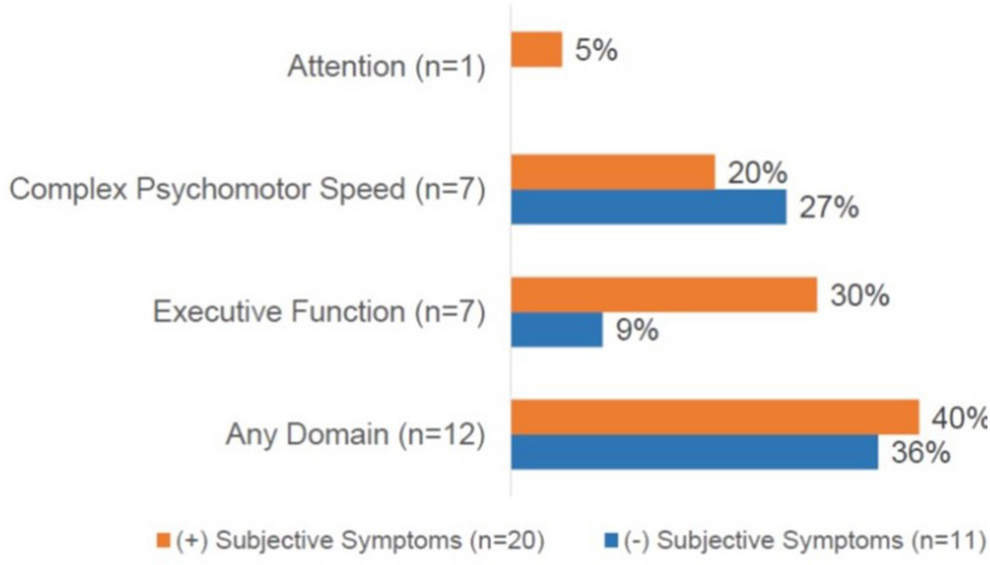
Cognitive domains impaired based on the presence of subjective dyscognition. A total of 12 patients (40%) had impairment on any cognitive domain. No impairments were demonstrated in the domains of general intellect or memory. CPT-3, The Conners continuous performance test, 3rd edition; D-KEFS, Delis-Kaplan executive function system; Grooved pegboard test assessesvisual-motor coordination. Subjects could demonstrate impairments in ≥1 domain.

Of the entire cohort, 39% (*n* = 12) demonstrated objective dyscognition. The presence of subjective dyscognition was not associated with objective dyscognition (Fisher's exact test, *p* = 0.29). The neurocognitive battery domains most impaired included complex psychomotor speed (23%), executive function (23%) and attention (3%) (Figure 2) and this was in the setting of good effort on the tests. The children with subjective dyscognition presented with greater anxiety, greater depression, lower resilience, lower health-related quality of life, and greater fatigue in comparison to the children without subjective dyscognition (Wilcoxon rank-sum test, all *p* < 0.05; Table 2). While it appeared that youth with subjective dyscognition had a longer pain duration (21 months [IQR: 12-26]) than those without subjective dyscognition (10 months [IQR: 5-60]) this was not statistically significant (*p* = 0.44) (Table 2).

We explored risk factors associated with the presence of subjective dyscognition. In bivariate logistic regression analysis, higher levels of functional disability (OR = 1.13, *p* = 0.02), depression (OR = 1.11, *p* = 0.02) and anxiety (OR = 1.09, *p* = 0.02) were associated with an increased odds of subjective dyscognition in the cohort, whereas lower HRQoL (OR = 0.82, *p* = 0.02), resilience (OR = 0.92, *p* = 0.03), and PedsQL fatigue scores (OR = 0.92, *p* = 0.01) were associated with a greater odds of subjective dyscognition (Table 4). We excluded depression (CDI-2) from the multivariable logistic regression analysis due to its multicollinearity with anxiety, resilience, and HRQoL measures. In a final multivariable logistic regression model resilience, functional disability and anxiety were found to be independently associated with subjective dyscognition (*p* < 0.05). Based on the OR estimates, with each increasing point on the functional disability inventory, the odds of subjective dyscognition increased by 19%; and similarly, for each increasing point on the MASC-2, the odds of anxiety increased by 12% (Table 4).

**Table 4:** Logistic regression predicting odds of subjective dyscognition.

	**Unadjusted ORs (95% CI)**	***P-*value**	**Adjusted ORs (95% CI)**	***P-*value**
**Patient-reported outcome measures (PROs)**				
Functional disability inventory (FDI) (0-60)	1.13 (1.02-1.26)	0.02^I^	1.19 (1.02-1.40)	0.03^I^
HRQoL (PROMIS global health 7 [PGH7])	0.82 (0.69-0.97)	0.02^I^	-	-
14-item resilience scale (14–98)	0.92 (0.85-0.99)	0.03^I^	-	-
CDI-2 (depression)	1.11 (1.02-1.22)	0.02^I^	-	-
MASC-2 (anxiety)	1.09 (1.02-1.17)	0.02^I^	1.12 (1.02-1.24)	0.02^I^
PedsQL general fatigue scale	0.92 (0.87-0.98)	0.01^I^	-	-
PedsQL sleep/rest fatigue scale	0.96 (0.91-1.00)	0.06	-	-

I *p-values suggesting statistical significance*.

*ORs, Odds Ratios; HRQoL, Health Related Quality of Life assessed using PROMIS (Patient Reported Outcome Measurement Information System) measures; CDI-2, The Children's Depression Inventory, 2nd Edition; MASC-2, The Multidimensional Anxiety Scale for Children, 2nd Edition; PedsQL, The Pediatric Quality of Life Inventory*.

*Depression was removed from the multivariable model due to multicolinearity. Backward selection method used with Stay Selection Level ≤ 0.15*.

## Discussion

In this cross-sectional study of adolescents with JFMS nearly two-thirds of patients reported subjective dyscognition and this was associated with functional disability and anxiety. The prevalence of subjective dyscognition was similar to what has been reported in the adult literature, with over 50% of adult patients with fibromyalgia (*n* = 57) experiencing dyscognition (1, 2). In our study, we found that 39% of teens experienced objective dyscognition, with impairments most prominently related to complex psychomotor speed, executive function, and attention. There was not, however, an association between subjective dyscognition and objective dyscognition identified on neuropsychological testing, suggesting that patient self-report of dyscognition may not be adequate in identifying neurocognitive impairment in this patient population.

Our findings are in line with previous research in adults with fibromyalgia syndrome suggesting that characteristic symptoms of the disease, such as depression and fatigue, have an impact on subjective dyscognition (3, 67–70). This relationship can be explained by the fact that affective and physical variables of fibromyalgia syndrome may exacerbate the amount of perceived effort required to perform the cognitive task, however, when confronted with the task, patients have the cognitive skills necessary to perform satisfactorily (9). Therefore, some affected patients may have elevated subjective cognitive deficits without compromising their objective performance on standardized testing. In the authors' experience, this is anecdotally reported by a number of teenagers with JFMS. They can excel academically despite symptoms of dyscognition but endorse that it requires significantly more effort than before the onset of their JFMS diagnosis or more effort than their peers require to succeed academically. Given the high prevalence of subjective dyscognition in patients with JFMS, interdisciplinary physical and psychosocial pain rehabilitation programs should help patients reframe their self-perceived deficits. Targeting resilience and other psychosocial risk factors through cognitive behavioral therapy holds tremendous value for this group of children (23, 51, 71, 72). Similarly, aerobic exercise has been found to have a positive impact on cognitive impairment in several chronic neurologic and psychiatric conditions and therefore may also lead to improvements in subjective dyscognition among youth with JFMS (73–76).

Our findings do suggest, however, that a significant proportion of youth with JFMS have objective neurocognitive deficits even in the setting of good effort. A little more than 1/3 of patients in our study demonstrated some degree of objective dyscognition that had not been previously diagnosed. While we did not include healthy adolescent controls in this study, the findings of objective dyscognition were reported based on normative data for each respective neuropsychological test and therefore are particularly concerning. Furthermore, to put this into the context of other rheumatic diseases, this is similar to the reported prevalence of cognitive dysfunction reported in systemic lupus erythematosus (ranging from 14 to 79%), which tends to affect executive functioning, visual organization and visual-motor functioning and psychomotor speed (77, 78). In contrast, children with juvenile idiopathic arthritis (JIA) have normal neurocognitive functioning (79, 80), unless pain is a predominant feature of their disease. In a study by Upadhyay et al. pain severity was associated with poorer cognitive function among youth with JIA (*n* = 16) (81). Therefore, additional research is needed to elucidate the neurologic, inflammatory, and other mechanistic underpinnings for these associations between pain and dyscognition. For now, our team recommends consideration of formal neuropsychological testing for youth with JFMS, especially those with subjective symptoms, as well as for youth with other chronic pain conditions.

While the source population for this study was from an outpatient setting staffed by pediatric rheumatologists, our findings have implications for physicians from all medical specialties and potentially impact clinical care across disciplines. First, given the limited workforce of pediatric rheumatologists (82–84), many teens with chronic widespread pain receive care from other specialists and our findings suggest that these providers may want to consider utilizing subjective measures of dyscognition in routine clinical care. Similarly, pain researchers might consider both subjective and objective measures of dyscognition as additional treatment outcomes to study in youth with chronic pain so that our findings can be compared to other pediatric chronic pain populations. Additionally, referrals for neuropsychological testing may be further considered in this patient population. Given the relatively high prevalence of dyscognition (both subjective and objective) in JFMS, providers caring for youth with chronic widespread pain along with symptoms of dyscognition may be more likely to consider whether their patient meets criteria for JFMS and provide referrals for non-pharmacologic multidisciplinary pain programs earlier on in the course of the patient's care.

This study has limitations. First, objective dyscognition was evaluated in a testing environment and not in a real-world setting (therefore having poor ecological validity) (68). Kratz et al. recently published a study assessing dyscognition in the lived environment among adults with fibromyalgia syndrome, compared to healthy controls (68). In this study, they found worse lab-based and ambulatory subjective and objective cognitive function for the fibromyalgia group compared to those without fibromyalgia, however, the authors did not adjust for distinguishing symptoms of fibromyalgia (e.g., pain, fatigue, and depression). Future work in JFMS should replicate this study design assessing dyscognition in one's everyday environment, especially within the school setting. Another limitation is the cross-sectional nature of this study. This study design prevented us from assessing intra-individual variations in cognitive functioning (68). However, we did adjust for symptom duration as well as factors indicative of the severity of one's fibromyalgia (including symptom severity and widespreadness of pain). Our understanding of intra-individual variations in subjective and objective dyscognition would benefit from future longitudinal study designs complemented by neuroimaging studies. The cross-sectional study design means that we cannot infer directionality or causality of our findings. Additionally, while the surveys administered in this study had varying scales and differed in their mode of delivery, there is nonetheless the possibility that common method variance could account for the associations between subjective dyscognition and other self-reported symptoms. Also, while we excluded from our study patients who may have been on stimulant medications for diagnosed attention deficits as a proxy measure for those subjects with pre-existing ADHD, we may not have entirely excluded all possible subjects with a known issue affecting cognitive processing and this may have contributed to our findings on objective dyscognition. Last, similar research needs to be conducted in other pediatric chronic pain populations to determine whether our findings are generalizable to pediatric chronic pain or specific to juvenile fibromyalgia syndrome.

One major strength of our study is its minimization of confounding factors. A benefit of our pediatric rheumatology division is that we emphasize a non-pharmacologic treatment approach for pediatric chronic pain. Therefore, we did not have to address the difficult challenge of monitoring medication intake for this study. Another strength is that we limited our inclusion criteria to youth with primary JFMS, minimizing their likelihood of having a co-morbid pain disorder that may have contributed to their cognitive abilities. However, these restrictive criteria are also responsible, in part, for our study's modest sample size.

## Conclusions

In summary, this study demonstrates that youth with JFMS experience both subjective and objective dyscognition but predominantly the former. Subjective dyscognition appears to be associated with symptoms of anxiety and overall functional disability. Providers managing teenagers with JFMS should consider routine use of self-reported measures of dyscognition in their clinical care as this is an important treatment outcome to consider. This work also has implications for the role of neuropsychologists, social work, and the educational system in facilitating continued academic success for patients with JFMS. Further work should examine dyscognition in JFMS longitudinally and in response to treatment with a combined physical and psychosocial pain rehabilitation program.

## Data Availability Statement

The raw data supporting the conclusions of this article will be made available by the authors, without undue reservation.

## Ethics Statement

The studies involving human participants were reviewed and approved by Institutional Review Board of the Children's Hospital of Philadelphia. Written informed consent to participate in this study was provided by the participants' legal guardian/next of kin.

## Author Contributions

SG, MS, PW, and JG were responsible for study design, data collection, and analyzation and interpretation of the findings. RX facilitated statistical plan, analysis of data, and interpretation of findings. EM facilitated subject recruitment, cleaning and maintenance of data, and provided critical feedback on the final manuscript. NT contributed to study data, data collection, and interpretation of the findings. JY and DS provided critical feedback and edits on the manuscript. All authors reviewed, critiqued, contributed to the manuscript and approved the final version.

## Funding

Research reported in this publication was supported and the National Center for Advancing Translational Sciences of the National Institutes of Health under award number UL1TR001878. This research was also funded by the Rheumatology Research Foundation.

## Author Disclaimer

The content is solely the responsibility of the authors and does not necessarily represent the official views of the National Institutes of Health.

## Conflict of Interest

The authors declare that the research was conducted in the absence of any commercial or financial relationships that could be construed as a potential conflict of interest.

## Publisher's Note

All claims expressed in this article are solely those of the authors and do not necessarily represent those of their affiliated organizations, or those of the publisher, the editors and the reviewers. Any product that may be evaluated in this article, or claim that may be made by its manufacturer, is not guaranteed or endorsed by the publisher.

## References

[1] Yunus M Masi AT Calabro JJ Miller KA Feigenbaum SL. Primary fibromyalgia (fibrositis): clinical study of 50 patients with matched normal controls. Semin Arthritis Rheum. (1981) 11:151–71. 10.1016/0049-0172(81)90096-2 6944796

[2] Katz RS Heard AR Mills M Leavitt F. The prevalence and clinical impact of reported cognitive difficulties (fibrofog) in patients with rheumatic disease with and without fibromyalgia. J Clin Rheumatol. (2004) 10:53–8. 10.1097/01.rhu.0000120895.20623.9f 17043464

[3] Kravitz HM Katz RS. Fibrofog and fibromyalgia: a narrative review and implications for clinical practice. Rheumatol Int. (2015) 35:1115–25. 10.1007/s00296-014-3208-7 25583051

[4] McAllister SJ Toussaint LL Williams DA Hoskin TL Whipple MO Vincent A. Perceived dyscognition reported by patients with fibromyalgia. Clin Exp Rheumatol. (2016) 34(2 Suppl 96):S48–54. 26941074

[5] Walitt B Ceko M Khatiwada M Gracely JL Rayhan R VanMeter JW. Characterizing “fibrofog”: subjective appraisal, objective performance, and task-related brain activity during a working memory task. NeuroImage Clin. (2016) 11:173–80. 10.1016/j.nicl.2016.01.021 26955513 PMC4761650

[6] Williamson J Larner AJ. Cognitive dysfunction in patients with fibromyalgia. Br J Hospital Med. (2016) 77:116. 10.12968/hmed.2016.77.2.116 26875810

[7] Pidal-Miranda M Gonzalez-Villar AJ Carrillo-de-la-Pena MT Andrade E Rodriguez-Salgado D. Broad cognitive complaints but subtle objective working memory impairment in fibromyalgia patients. PeerJ. (2018) 6:e5907. 10.7717/peerj.5907 30498630 PMC6252063

[8] Glass JM. Review of cognitive dysfunction in fibromyalgia: a convergence on working memory and attentional control impairments. Rheum Dis Clin North Am. (2009) 35:299–311. 10.1016/j.rdc.2009.06.002 19647144

[9] Bar-On Kalfon T Gal G Shorer R Ablin JN. Cognitive functioning in fibromyalgia: the central role of effort. J Psychosom Res. (2016) 87:30–6. 10.1016/j.jpsychores.2016.06.004 27411749

[10] Verdejo-Garcia A Lopez-Torrecillas F Calandre EP Delgado-Rodriguez A Bechara A. Executive function and decision-making in women with fibromyalgia. Arch Clin Neuropsychol. (2009) 24:113–22. 10.1093/arclin/acp014 19395361

[11] Mease P Arnold LM Choy EH Clauw DJ Crofford LJ Glass JM. Fibromyalgia syndrome module at OMERACT 9: domain construct. J Rheumatol. (2009) 36:2318–29. 10.3899/jrheum.090367 19820221 PMC3419373

[12] Berger A Sadosky A Dukes EM Edelsberg J Zlateva G Oster G. Patterns of healthcare utilization and cost in patients with newly diagnosed fibromyalgia. Am J Managed Care. (2010) 16(5 Suppl):S126-37. 20586521

[13] Kaufman EL Tress J Sherry DD. Trends in medicalization of children with amplified musculoskeletal pain syndrome. Pain Med. (2017) 18:825–31. 10.1093/pm/pnw188 27497319

[14] Tian F Guittar P Bout-Tabaku SM. Chronic pain in children seen at a rheumatology clinic: healthcare utilization patterns. Arthritis Rheumatol. (2016) 68 (suppl 10). Available online at: https://acrabstracts.org/abstract/chronic-pain-in-children-seen-at-a-rheumatology-clinic-healthcare-utilization-patterns/ (accessed February 10, 2022).

[15] Murray CB Groenewald CB de la Vega R Palermo TM. Long-term impact of adolescent chronic pain on young adult educational, vocational, and social outcomes. Pain. (2020) 161:439–45. 10.1097/j.pain.0000000000001732 31651579 PMC7001863

[16] Olsen MN Sherry DD Boyne K McCue R Gallagher PR Brooks LJ. Relationship between sleep and pain in adolescents with juvenile primary fibromyalgia syndrome. Sleep. (2013) 36:509–16. 10.5665/sleep.2534 23564998 PMC3612246

[17] Hoffart C Fortney S Wallace D. Sleep disruption in children with chronic pain. Arthritis Rheumatol. (2016) 68 (suppl 10). Available online at: https://acrabstracts.org/abstract/sleep-disruption-in-children-with-chronic-pain/ (accessed February 10, 2022).

[18] Molina J Dos Santos FH Terreri MTRA Fraga MM Silva SG Hilário MOE. Sleep, stress, neurocognitive profile and health-related quality of life in adolescents with idiopathic musculoskeletal pain. Clinics. (2012) 67:1139–44. 10.6061/clinics/2012(10)04 23070339 PMC3460015

[19] Wolfe F Clauw DJ Fitzcharles MA Goldenberg DL Katz RS Mease P. The American College of Rheumatology preliminary diagnostic criteria for fibromyalgia and measurement of symptom severity. Arthritis Care Res. (2010) 62:600–10. 10.1002/acr.20140 20461783

[20] Walker LS Greene JW. The functional disability inventory: measuring a neglected dimension of child health status. J Pediatr Psychol. (1991) 16:39–58. 10.1093/jpepsy/16.1.39 1826329

[21] Kashikar-Zuck S Flowers SR Claar RL Guite JW Logan DE Lynch-Jordan AM. Clinical utility and validity of the Functional Disability Inventory (FDI) among a multicenter sample of youth with chronic pain. Pain. (2011) 152:1600–7. 10.1016/j.pain.2011.02.050 21458162 PMC3114262

[22] Claar RL Walker LS. Functional assessment of pediatric pain patients: psychometric properties of the functional disability inventory. Pain. (2006) 121:77–84. 10.1016/j.pain.2005.12.002 16480823 PMC3144698

[23] Sil S Arnold LM Lynch-Jordan A Ting TV Peugh J Cunningham N. Identifying treatment responders and predictors of improvement after cognitive-behavioral therapy for juvenile fibromyalgia. Pain. (2014) 155:1206–12. 10.1016/j.pain.2014.03.005 24650858 PMC4058411

[24] Flowers SR Kashikar-Zuck S. Measures of juvenile fibromyalgia: functional disability inventory (FDI), modified fibromyalgia impact questionnaire-child version (MFIQ-C), and pediatric quality of life inventory (PedsQL) 3.0 rheumatology module pain and hurt scale. Arthritis Care Res. (2011) 63(Suppl 11):S431–7. 10.1002/acr.20639 22588763 PMC3716003

[25] McGrath PA Gillespie J. Pain assessment in children and adolescents. In: Turk DC Melzack R, editors. Handbook of Pain Assessment. New York, NY: The Guildford Press (2001). p. 97–118.

[26] Salaffi F Stancati A Silvestri CA Ciapetti A Grassi W. Minimal clinically important changes in chronic musculoskeletal pain intensity measured on a numerical rating scale. Eur J Pain. (2004) 8:283–91. 10.1016/j.ejpain.2003.09.004 15207508

[27] Wolfe F Smythe HA Yunus MB Bennett RM Bombardier C Goldenberg DL. The American College of Rheumatology 1990 criteria for the classification of Fibromyalgia. Report of the multicenter criteria committee. Arthritis Rheum. (1990) 33:160–72. 10.1002/art.1780330203 2306288

[28] Wolfe F Clauw DJ Fitzcharles MA Goldenberg DL Hauser W Katz RL. 2016 Revisions to the 2010/2011 fibromyalgia diagnostic criteria. Semin Arthritis Rheum. (2016) 46:319–29. 10.1016/j.semarthrit.2016.08.012 27916278

[29] Ting TV Barnett K Lynch-Jordan A Whitacre C Henrickson M Kashikar-Zuck S. 2010 American College of Rheumatology Adult fibromyalgia criteria for use in an adolescent female population with juvenile fibromyalgia. J Pediatr. (2016) 169:181–7.e1. 10.1016/j.jpeds.2015.10.011 26545727 PMC7675923

[30] De Sanctis V Abbasciano V Soliman AT Soliman N Di Maio S Fiscina B. The juvenile fibromyalgia syndrome (JFMS): a poorly defined disorder. Acta Bio Med. (2019) 90:134–48. 10.23750/abm.v90i1.8141 30889168 PMC6502146

[31] Forrest CB Bevans KB Pratiwadi R Moon J Teneralli RE Minton JM. Development of the PROMIS (R) pediatric global health (PGH-7) measure. Qual Life Res. (2014) 23:1221–31. 10.1007/s11136-013-0581-8 24264804 PMC3966936

[32] Forrest CB Tucker CA Ravens-Sieberer U Pratiwadi R Moon J Teneralli RE. Concurrent validity of the PROMIS(R) pediatric global health measure. Qual Life Res. (2016) 25:739–51. 10.1007/s11136-015-1111-7 26310283

[33] Thissen D Liu Y Magnus B Quinn H Gipson DS Dampier C. Estimating minimally important difference (MID) in PROMIS pediatric measures using the scale-judgment method. Qual Life Res. (2016) 25:13–23. 10.1007/s11136-015-1058-8 26118768 PMC4695321

[34] Kovacs M. Children's depression inventory (CDI and CDI 2). In: Cautin RL Lilienfeld SO, editors. The Encyclopedia of Clinical Psychology. John Wiley & Sons, Inc. (2014).

[35] Wei C Hoff A Villabø MA Peterman J Kendall PC Piacentini J. Assessing anxiety in youth with the multidimensional anxiety scale for children (MASC). J Clin Child Adolesc Psychol. (2014) 43:566–78. 10.1080/15374416.2013.814541 23845036 PMC3858516

[36] March JS Parker JD Sullivan K Stallings P Conners CK. The Multidimensional Anxiety Scale for Children (MASC): factor structure, reliability, and validity. J Am Acad Child Adolesc Psychiatry. (1997) 36:554–65. 10.1097/00004583-199704000-00019 9100431

[37] March JS Sullivan K. Test-retest reliability of the multidimensional anxiety scale for children. J Anxiety Disord. (1999) 13:349–58. 10.1016/S0887-6185(99)00009-2 10504106

[38] Gioia GA Insquith PK Guy SC Kenworthy L. Behavior Rating Inventory of Executive Function, Second Edition (BRIEF-2) PAR. Lutz, FL: Second Psychological Assessment Resources. (2015).

[39] Varni JW Limbers CA. The PedsQL multidimensional fatigue scale in young adults: feasibility, reliability and validity in a University student population. Qual Life Res. (2008) 17:105–14. 10.1007/s11136-007-9282-5 18027106

[40] Panepinto JA Torres S Bendo CB McCavit TL Dinu B Sherman-Bien S. PedsQL™ multidimensional fatigue scale in sickle cell disease: feasibility, reliability, and validity. Pediatr Blood Cancer. (2014) 61:171–7. 10.1002/pbc.24776 24038960 PMC3848797

[41] Varni JW Burwinkle TM Katz ER Meeske K Dickinson P. The PedsQL in pediatric cancer: reliability and validity of the pediatric quality of life inventory generic core scales, multidimensional fatigue scale, and cancer module. Cancer. (2002) 94:2090–106. 10.1002/cncr.10428 11932914

[42] Varni JW Beaujean A Limbers CA. Factorial invariance of pediatric patient self-reported fatigue across age and gender: a multigroup confirmatory factor analysis approach utilizing the PedsQL™ multidimensional fatigue scale. Qual Life Res. (2013) 22:2581–94. 23423759 10.1007/s11136-013-0370-4

[43] Varni JW Burwinkle TM Szer IS. The PedsQL multidimensional fatigue scale in pediatric rheumatology: reliability and validity. J Rheumatol. (2004) 31:2494–500. 15570657

[44] Limbers C Young D Jernigan S Bryant W Stephen M. Comparison between objective measures and parental behavioral rating scales of memory and attention in pediatric endocrinology patients. Appl Neuropsychol Child. (2016) 2016:1–8. 10.1080/21622965.2016.1152892 27183244

[45] Varni JW Limbers CA Sorensen LG Neighbors K Martz K Bucuvalas JC. PedsQL™ cognitive functioning scale in pediatric liver transplant recipients: feasibility, reliability and validity. Qual Life Res. (2011) 20:913–21. 10.1007/s11136-010-9823-1 21184184 PMC3763499

[46] Pritzker S Minter A. Measuring adolescent resilience: an exmaination of the cross-ethnic validity of the RS-14. Children Youth Serv Rev. (2014) 44:328–33. 10.1016/j.childyouth.2014.06.022

[47] Wagnild G. The Resilience Scale User's Guide for the US English version of the Resilience Scale and the 14-Item Resilience Scale (RS-14) (2009).

[48] Wagnild G. A review of the resilience scale. J Nurs Measurement. (2009) 17:105–13. 10.1891/1061-3749.17.2.105 19711709

[49] Wagnild GM Young HM. Development and psychometric evaluation of the Resilience Scale. J Nurs Measurement. (1993) 1:165–78. 7850498

[50] Winsett RP Stender SR Gower G Burghen GA. Adolescent self-efficacy and resilience in participants attending A diabetes camp. Pediatr Nurs. (2010) 36:293–6; quiz 7. 21291045

[51] Gmuca S Xiao R Urquhart A Weiss PF Gillham JE Ginsburg KR. The role of patient and parental resilience in adolescents with chronic musculoskeletal pain. J Pediatr. (2019) 210:118–26.e2. 10.1016/j.jpeds.2019.03.006 30981421

[52] Wechsler D. Wechsler Abbreviated Scale of Intelligence, Second Edition (WASI-II). San Antonio, TX: NCS Pearson (2011).

[53] Wechsler D. Wechsler Abbreviated Scale of Intelligence. New York, NY: The Psychological Corporation: Harcourt Brace & Company (1999).

[54] Piehl JJ Wolff M Hahm J. Test review of the child and adolescent memory profile (ChAMP). J Pediatr Neuropsychol. (2016) 2016:1–5. 10.1007/s40817-016-0023-y

[55] Watkins MW. Structure of the Wechsler Intelligence Scale for Children–Fourth Edition among a national sample of referred students. Psychol Assess. (2010) 22:782–7. 10.1037/a0020043 21133545

[56] Wechsler D. Wechsler Intelligence Scale for Children, 5th Edition (WISC-V). San Antonio, TX: Pearson PsychCorp (2014).

[57] Wechsler D. Wechsler Adult Intelligence Scale-Fourth Edition (WAIS-IV). San Antonio, Tx: Pearson PsychCorp (2008).

[58] Conners CK. Conners Continuous Performance Test- Third Edition (Conners CPT 3) & Conners Continuous Auditory Test of Attention (Conners CATA): Technical Manual. New York, NY: Multi-Health Systems Inc. (2014).

[59] Homack S Lee D Riccio CA. Test review: Delis-Kaplan executive function system. J Clin Exp Neuropsychol. (2005) 27:599–609. 10.1080/13803390490918444 16019636

[60] Delis DC Kaplan E Kramer J. Delis Kaplan Executive Function System. San Antonio, TX: The Psychological Corporation (2001).

[61] Cherry BJ Zettel-Watson L Shimizu R Roberson I Rutledge DN Jones CJ. Cognitive performance in women aged 50 years and older with and without fibromyalgia. J Gerontol Series B, Psychol Sci Soc Sci. (2014) 69:199–208. 10.1093/geronb/gbs122 23275498 PMC3976117

[62] Lezak MD Howieson DB Loring DW. Neuropsychological Assessment. 4th ed. New York, NY: Oxford University Press (2004).

[63] Mitrushina MN Boone KB D'Elia LF. Handbook of Normative Data for Neuropsychological Assessment. 2nd ed. New York, NY: Oxford University Press (2005).

[64] Sutton GP Barchard KA Bello DT Thaler NS Ringdahl E Mayfield J. Beery-Buktenica Developemental Test of visual-motor integration performance in childrne with traumatic brain injury and attention-deficit/hyperactivity disorder. Psychol Assess. (2011) 23:3. 10.1037/a0023370 21875221

[65] Kirkwood MW Yeates KO Randolph C Kirk JW. The implications of symptom validity test failure for ability-based test performance in a pediatric sample. Psychol Assess. (2012) 24:36–45. 10.1037/a0024628 21767023

[66] Green P. Green's Medical Symptom Validity Test (MSVT) for Microsoft Windows: User's Manual. Edmonton, Canada: Green's Publising (2004).

[67] Suhr JA. Neuropsychological impairment in fibromyalgia: relation to depression, fatigue, and pain. J Psychosom Res. (2003) 55:321–9. 10.1016/S0022-3999(02)00628-1 14507543

[68] Kratz AL Whibley D Kim S Sliwinski M Clauw D Williams DA. Fibrofog in daily life: An examination of ambulatory subjective and objective cognitive function in fibromyalgia. Arthritis Care Res. (2019) 72:1669–77. 10.1002/acr.24089 31609548 PMC7153985

[69] Gelonch O Garolera M Valls J Rossello L Pifarre J. Executive function in fibromyalgia: comparing subjective and objective measures. Comprehens Psychiatry. (2016) 66:113–22. 10.1016/j.comppsych.2016.01.002 26995244

[70] Gelonch O Garolera M Valls J Castella G Varela O Rossello L. The effect of depressive symptoms on cognition in patients with fibromyalgia. PLoS ONE. (2018) 13:e0200057. 10.1371/journal.pone.0200057 29975749 PMC6033429

[71] Kashikar-Zuck S Black WR Pfeiffer M Peugh J Williams SE Ting TV. Pilot randomized trial of integrated cognitive-behavioral therapy and neuromuscular training for juvenile fibromyalgia: the FIT Teens Program. J Pain. (2018) 19:1049–62. 10.1016/j.jpain.2018.04.003 29678563 PMC6119635

[72] Kashikar-Zuck S Parkins IS Graham TB Lynch AM Passo M Johnston M. Anxiety, mood, and behavioral disorders among pediatric patients with juvenile fibromyalgia syndrome. Clin J Pain. (2008) 24:620–6. 10.1097/AJP.0b013e31816d7d23 18716501 PMC3711138

[73] Northey JM Cherbuin N Pumpa KL Smee DJ Rattray B. Exercise interventions for cognitive function in adults older than 50: a systematic review with meta-analysis. Br J Sports Med. (2018) 52:154–60. 10.1136/bjsports-2016-096587 28438770

[74] da Silva FC Iop RDR de Oliveira LC Boll AM de Alvarenga JGS Gutierres Filho PJB. Effects of physical exercise programs on cognitive function in Parkinson's disease patients: a systematic review of randomized controlled trials of the last 10 years. PLoS ONE. (2018) 13:e0193113. 10.1371/journal.pone.0193113 29486000 PMC5828448

[75] Liu IT Lee WJ Lin SY Chang ST Kao CL Cheng YY. The therapeutic effects of exercise training on elderly patients with dementia: a randomized controlled trial. Arch Phys Med Rehabil. (2020) 101:762–9. 10.1016/j.apmr.2020.01.012 32084347

[76] Shimada T Ito S Makabe A Yamanushi A Takenaka A Kawano K. Aerobic exercise and cognitive functioning in schizophrenia: results of a 1-year follow-up from a randomized controlled trial. Psychiatry Res. (2020) 286:112854. 10.1016/j.psychres.2020.112854 32078891

[77] Rayes HA Tani C Kwan A Marzouk S Colosimo K Medina-Rosas J. What is the prevalence of cognitive impairment in lupus and which instruments are used to measure it? A systematic review and meta-analysis. Semin Arthritis Rheum. (2018) 48:240–55. 10.1016/j.semarthrit.2018.02.007 29571540

[78] AlE'ed A Vega-Fernandez P Muscal E Hinze CH Tucker LB Appenzeller S. Challenges of diagnosing cognitive dysfunction with neuropsychiatric systemic lupus erythematosus in childhood. Arthritis Care Res. (2017) 69:1449–59. 10.1002/acr.23163 27992660 PMC5476521

[79] Granjon M Rohmer O Doignon-Camus N Popa-Roch M Pietrement C Gavens N. Neuropsychological functioning and academic abilities in patients with juvenile idiopathic arthritis. Pediatr Rheum Online J. (2021) 19:53. 10.1186/s12969-021-00541-1 33853628 PMC8048299

[80] Feldmann R Weglage J Roth J Foell D Frosch M. Systemic juvenile rheumatoid arthritis: cognitive function and social adjustment. Ann Neurol. (2005) 58:605–9. 10.1002/ana.20626 16178013

[81] Upadhyay J Lemme J Cay M Van Der Heijden H Sibai D Goodlett B. A multidisciplinary assessment of pain in juvenile idiopathic arthritis. Semin Arthritis Rheum. (2021) 51:700–11. 10.1016/j.semarthrit.2021.05.011 34139523 PMC9741862

[82] Henrickson M. Policy challenges for the pediatric rheumatology workforce: part I. Education and economics. Pediatr Rheumatol Online J. (2011) 9:23. 10.1186/1546-0096-9-24 21846336 PMC3170606

[83] Foster H Rapley T. Access to pediatric rheumatology care – a major challenge to improving outcome in juvenile idiopathic arthritis. J Rheumatol. (2010) 37:2199–202. 10.3899/jrheum.100910 21041261

[84] Foster HE Scott C Tiderius CJ Dobbs MB. The paediatric global musculoskeletal task force - towards better MSK health for all. Pediatr Rheumatol Online J. (2020) 18:60. 10.1186/s12969-020-00451-8 32664961 PMC7359433

